# Providing guidance for genomics-based cancer treatment decisions: insights from stakeholder engagement for post-prostatectomy radiation therapy

**DOI:** 10.1186/s12911-017-0526-1

**Published:** 2017-08-24

**Authors:** James Abe, Jennifer M. Lobo, Daniel M. Trifiletti, Timothy N. Showalter

**Affiliations:** 1 Department of Radiation Oncology, University of Virginia School of Medicine, 1240 Lee Street, Box 800383, Charlottesville, VA 22908 USA; 2 Department of Public Health Sciences, University of Virginia School of Medicine, Charlottesville, VA USA

**Keywords:** Genomics, Genomic-driven medicine, Personalized medicine, Prostate cancer, Adjuvant radiation therapy, Stakeholder engagement

## Abstract

**Background:**

Despite the emergence of genomics-based risk prediction tools in oncology, there is not yet an established framework for communication of test results to cancer patients to support shared decision-making. We report findings from a stakeholder engagement program that aimed to develop a framework for using Markov models with individualized model inputs, including genomics-based estimates of cancer recurrence probability, to generate personalized decision aids for prostate cancer patients faced with radiation therapy treatment decisions after prostatectomy.

**Methods:**

We engaged a total of 22 stakeholders, including: prostate cancer patients, urological surgeons, radiation oncologists, genomic testing industry representatives, and biomedical informatics faculty. Slides were at each meeting to provide background information regarding the analytical framework. Participants were invited to provide feedback during the meeting, including revising the overall project aims. Stakeholder meeting content was reviewed and summarized by stakeholder group and by theme.

**Results:**

The majority of stakeholder suggestions focused on aspects of decision aid design and formatting. Stakeholders were enthusiastic about the potential value of using decision analysis modeling with personalized model inputs for cancer recurrence risk, as well as competing risks from age and comorbidities, to generate a patient-centered tool to assist decision-making. Stakeholders did not view privacy considerations as a major barrier to the proposed decision aid program. A common theme was that decision aids should be portable across multiple platforms (electronic and paper), should allow for interaction by the user to adjust model inputs iteratively, and available to patients both before and during consult appointments. Emphasis was placed on the challenge of explaining the model’s composite result of quality-adjusted life years.

**Conclusions:**

A range of stakeholders provided valuable insights regarding the design of a personalized decision aid program, based upon Markov modeling with individualized model inputs, to provide a patient-centered framework to support for genomic-based treatment decisions for cancer patients. The guidance provided by our stakeholders may be broadly applicable to the communication of genomic test results to patients in a patient-centered fashion that supports effective shared decision-making that represents a spectrum of personal factors such as age, medical comorbidities, and individual priorities and values.

**Electronic supplementary material:**

The online version of this article (10.1186/s12911-017-0526-1) contains supplementary material, which is available to authorized users.

## Background

Despite exciting advances toward the promise of genomics-driven cancer treatment [1, 2], there is not yet an established framework for optimal communication of test results in a clinical setting in a way that cancer best informs decision-making [3]. Research on the implementation of gene expression profiling for cancer treatment decisions has identified variable levels of understanding among patients, with misperceptions of test validity [4], and concerns among oncologists regarding patients’ understanding of test results [5]. This suggests a need for decision aids to support communication of genomic expression profiling test results and informed decision-making. Optimal implementation of genomic risk stratification tools, which have the potential to better match patients with the right treatments for them, should be performed within a patient-centered context that meets the National Academy of Medicine (formerly Institute of Medicine) goals for patient-centeredness by considering “individual patient preferences, needs, and values, and ensuring that patient values guide all clinical decisions” [6, 7]. As an example, the current study involves the clinical situation of prostate cancer patients faced with decisions regarding postoperative radiation therapy (RT) decisions, where the consideration of genomic test-defined individualized cancer recurrence estimates may be implemented within a patient-centered shared decision-making model.

Many of the nearly half of patients with localized prostate cancer who undergo radical prostatectomy (RP) will either develop a biochemical recurrence, in the form of a rising prostate specific antigen (PSA) blood level, or will be considered to be at high risk of recurrence based on adverse pathological features [8–10]. These men will face treatment decisions regarding the use of adjuvant RT (ART) after RP and/or close biochemical observation with salvage RT (SRT) for PSA recurrence. ART has been shown to improved outcomes compared to observation for high-risk patients [11–13], but close observation with serial PSA testing and selective use of SRT is also an effective treatment strategy [14] that has the potential advantage of limits the total number of patients exposed to the risks of radiation-related toxicities [15]. Currently, there is a relative lack of comparative data to evaluate the relative benefits of ART versus SRT, and joint guidelines from the American Urological Association (AUA) and the American Society for Radiation Oncology (ASTRO) recommend that clinicians presents both ART and SRT as reasonable treatment options and counsel patients appropriately for shared decision-making [16].

Decisions regarding ART and SRT after RP are complex for patients and clinicians, since choices can be influenced by an individual’s recurrence risk, medical comorbidities and personal preferences and values. A recent Markov decision analysis model considering length and quality of life for ART versus SRT showed that the preferred treatment strategy for an individual patient is dependent upon the specific probabilities for cancer recurrence, as defined based on a genomic risk classifier assay, as well as death from causes other than prostate cancer [17]. Genomic predictors of prostate cancer outcomes are increasingly available for clinical use [18, 19], including the Decipher® genomic classifier (GC) test (GenomeDx Biosciences, San Diego, CA) that provides individual estimates of the risk of metastasis after RP and is intended to be used for decision-making regarding ART and SRT [20, 21]. Although the information provided by the GC test has been shown to influence the recommendations of clinicians for ART after prostatectomy [22, 23], GC test results can be complex and it is not yet clear how patients and their families will incorporate the genomic-based risk estimates into treatment decisions.

In the current report, we describe a stakeholder engagement program that we performed in order to develop a framework for individualized decision aids to assist in the shared decision making process for patients making ART and/or SRT decisions with individualized GC-based recurrence risk estimates. Stakeholder engagement is a critical process for the development and implementation of genomic tests for clinical decision-making, because various stakeholders may each have different perspectives on genomic information [24]. We evaluate findings from the stakeholder engagement process completed prior to development of a decision aid tool that would consider an individual patient’s age, genomic-based risk of cancer recurrence, and other inputs to provide personalized guidance for post-RP ART and SRT decisions. Although our long-term objective is to develop a personalized decision aid program based upon a decision analysis modeling approach that incorporates individualized model inputs, we engaged a diverse group of stakeholders at an early point in the conceptual development of the proposed decision intervention with the goal of influencing the direction and format of the decision aid program to create a practical, patient-centered tool.

## Methods

Our stakeholder meeting interview team was comprised of two faculty investigators (TNS and JML) with prior research experience and training in stakeholder engagement. Stakeholders were identified and recruited through direct contact with the research team. Patient stakeholders were identified in a single radiation oncology clinic by their physician (TNS), while other stakeholder groups were identified through informal networking by email and at medical conferences. The team conducted a series of meetings with stakeholders to inform the process of developing a personalized decision aid program.

Each stakeholder meeting with investigators included only one class of stakeholders. For example, either prostate cancer patients or urologists, but not both, would be invited to each meeting. This approach was used in order to encourage participation and avoid imbalance in power or educational level between groups. Although this choice may have improved convenience and facilitated conversation, the lack of larger focus group may have limited the range of information obtained from the process. A prepared set of slides was used to kick-off each meeting and provide background information (see Additional file 1). Much of the background information was based upon recent research using Markov decision analysis modeling that demonstrated that treatment choices that maximized length and quality of life for individual patients are dependent upon GC-based recurrence risk and risk of mortality from causes other than prostate cancer [17]. Patient stakeholder meetings occurred with individual patients and the investigators, mostly to preserve patient privacy and to maximize convenience for participants by scheduling interviews on the same day as clinical appointments, but also to stay on topic and to avoid the risk of unfocused or tangential discussions that can be observed with larger focus groups [25].

Each participant was invited to provide feedback during the meeting, including opportunities to revise the overall project aims. No specific time limit was applied to the stakeholder meetings, but the discussions lasted 25–45 min based on the amount of feedback and discussion offered by participants. Semi-structured interview structure was followed with prompts from a standardized data collection form that provided interviewers potential questions. For example: “What are the most important issues that you would consider for this decision?”; “What evidence do you need to make a treatment decision?”; “What format would you prefer for information?”; “Where would you prefer to receive information to help with a treatment decision?”; and, “Who would you like to have with you during the process of receiving information?”. The interview also included open-ended questions such as: “How would you design this differently?”; “Do you have additional ideas regarding this topic?”; and “Do you have additional ideas regarding how you would feel about using genomic tests for decisions?”. No quantitative data were collected. Audio and/or video recording of the meeting was not performed. The interviewers transcribed content from the meetings immediately upon completion of the meetings. The study team informed participants regarding the intention to preserve anonymity and to avoid specific attribution of quotes to individual participants in order to facilitate candid discussion.

### Stakeholder groups

There were a total of 5 stakeholder groups included in the study: patients with prostate cancer (5 participants), urological surgeons (5 participants), radiation oncologists (5 participants), genomic testing industry representatives (5 participants), and biomedical informatics faculty members (2 participants). The stakeholder participants were chosen for their experience within their respective group. The industry representatives were all employees of GenomeDx, the company that markets the Decipher GC test. The IRB approval of the stakeholder engagement protocol permitted participation of up to 15 prostate cancer patients, but this was limited to 5 patients after investigators noted that response content had been exhausted with no new themes identified after the initial 3 patient stakeholder sessions.

### Meeting preparation

Prior to each meeting, a common slide deck was shared with participants (see Additional file 1). At the start of each meeting, the study team first presented the slides, allowing time for questions and discussion, to provide background information. The materials provided included an overview of stakeholder engagement, general clinical context regarding ART and SRT decision-making for prostate cancer, background and results for our group’s Markov modeling approach using individualized model inputs for ART versus SRT after RP [17], and a general proposed framework for a patient-centered decision aid based that incorporated individual model inputs to include at least GC test results and risk of death from causes other than prostate cancer. The materials were intended to provide the above information at a high school reading level. The investigators based the proposed decision aid heuristic model on the Markov modeling methods in order to provide an analytic framework that could synthesize a range of clinical outcomes into summary model outputs.

### Analysis

Transcribed data was collected from each stakeholder meeting. In the event of the interview team members having different content transcribed, an inclusive approach was taken with inclusion of all transcribed content and statements. Stakeholder meeting content was reviewed and the content was summarized by stakeholder group according to themes. Since the semi-structured interviews permitted open-ended discussion, providing a broad range of content, our synthesis of content into themes focused on presenting the broadest spectrum of content possible. Quantitative analysis was not performed.

## Results

A total of 22 stakeholders participated in the meetings. Among the 5 patient stakeholders, all participants held college degrees and 3 participants held graduate degrees, reflecting the patient population at our institution. Most physician stakeholders were male (9 of 10), with one female urologist included. Stakeholders from the genomic testing industry were all employees of GenomeDx.

Stakeholders offered comments along a range of topics and perspectives. Stakeholder content was categorized according to 4 subjects: empathy, privacy concerns, design and formatting, and location or context of decision aid delivery to patient (Table 1). The majority of suggestions from all stakeholder groups focused on aspects of decision aid design and formatting, as well as delivery location/context and access to the decision aid, with the least time in discussion focusing privacy concerns. In general, stakeholders indicated that privacy considerations did not present significant barriers to the proposed decision aid framework, partly due to an understood distinction between tumor genomics versus an individual patient’s germline genetic information. Regarding location and context of decision aid delivery, a common theme was that stakeholders felt information should be disseminated across multiple platforms and should be available both before and during consult appointments. Regarding design, stakeholders consistently emphasized the need for simple graphs and figures and to remove any jargon and acronyms that might confuse patients. Stakeholders consistently recommended that only individualized data should be featured in the primary report, with the goal of showing patients and providers personalized information without the added complexity of a side-by-side comparison with average numbers. Emphasis was placed on the need to address the challenge of explaining quality-adjusted life years (QALYs) – a numerical measure of disease burden that incorporates length and quality of life – in decision aid materials, since the analytic framework of the personalized reports is based upon a Markov modeling approach that using individualized inputs [17]. It was recommended that the decision aid format be interactive, with patients being able to iteratively adjust model inputs to see the impact on model outcomes.

**Table 1:** Summary of content from stakeholder meetings regarding development of personalized decision aids to guide genomics-informed treatment decisions for radiation therapy after prostatectomy

Stakeholder Group	Thematic Category			
	Empathy	Privacy Concerns	Formatting	Location/Access
Patients (*n* = 5)	- This is a time of high anxiety, so it is hard to absorb data. A few bullet points to help address anxiety important. - There is appreciation that patient has to make decision. This helps guide discussion. - This helps frame decision. There are some slow deciders, and this helps them be better at managing anxiety. - From genomics standpoint, there is always fear regarding genetics and insurance companies—need to address how this differs from genetics. - This program makes the genomic test more human. - For mortality risk input, be sure not to scare patients. Consider putting behind the scenes. Just focus on prostate cancer for patients, hide the rest.	- For websites, concerns about privacy. Anonymizing the process would help. - Offering a CD or thumb drive might help with feeling of privacy. - I have no concerns about genomics and privacy, since this is different than genetic risks.	- Need summary page for main points, plus option of seeing more details. - Recommend showing “spread disease” as an image to teach what metastasis is. - Average value comparison (vs. individualized results) not important, doesn’t belong in main document for patients. - Life expectancy predictions should include more elements than just age. - Be sure to spell out acronyms. - Graphs and numbers both important, because patients decide differently. - Suggest consider showing QALYs vertically and include width representation of percentage of cases where this recommendation is preferred by the model. So, the overall area of the box shows how much “better” the option is. - Like the idea of using graphs to present personalized outcome. - Need to make this interactive with ability to change inputs/assumptions. Want to be able to make thoughtful adjustments to personalized inputs. - Utilities for prostate cancer outcomes may not be the same for surgery patients as for all patients. - Would like ability to change variables as much as possible in program to see the effects of each number. - Recommend combining charts together as much as possible—maybe include the average results bar overlaid within same graph. - Area chart looks good, or consider stacked bar graph since that is height only - This program helps to show factors that affect decision; more info better always - Make the distinction between human genome and cancer genome. - Consider adding exercise and lifestyle factors as option to influence mortality estimates, as this could be another opportunity to influence lifestyle choices	- Information should be available in office for review with physicians. -This should be available WHEREVER THE PATIENT WANTS IT. - Be sure to categorize and thematically link all information so it is easy to understand. - I like to be pre-informed, so would want this tool first prior to urology appointment. Then, can bring questions/prepare. - Really need to do this early before the meeting with doctor. This is helpful, but would be overwhelming without time to prepare. - Would want MD to present this tool, but then give some space to consider the information.
Urologists (*n* = 5)	- May be of interest to some patients; this will reassure some anxious patients.	None.	- Patients like numbers and figures, so this will help to facilitate discussion. - Urinary, fecal and sexual side effects important for patients, so should try to include estimated risks. - Really have to explain QALYs and life years well. - Need to communication rate of developing metastatic disease, and that that is linked to receiving ADT. - Data incorporated is similar to nomograms in use in clinic, so there is precedent. - Simple graphical display of survival and complications curves best. - Data regarding impact of predictive data on treatment outcomes are needed to best inform the model. - The most important drivers of clinical decision are data regarding ART versus very early SRT with ultrasensitive PSA, and there are limited such data to drive the model for this decision aid. - Genomic information plus risks of therapy are the most important drivers for the clinical decision	- This data should be delivered in clinic with doctor. - Potential stumbling blocks for this comes down to execution and usability/access of the program. - Genomics seems more like black box to many, compared to other clinical factors, so patients will depend on urologists for information. - This provides a good framework for the clinical decision. - Recommend focusing on iPad/iPhone format for access and portability. - Reality is that most community docs refer to larger center, where this would be discussed. - Most patients (80%) would like this. - This is most useful in intermediate risk patients. - This should be delivered 1:1 with patients. This may fit well in multidisciplinary clinic where it will help build consensus over time.
Radiation Oncologists (*n* = 5)	None.	None.	- This decision aid will have value in clinic. For physicians and patients alike, a web based tool would be attractive similar to the MSKCC nomogram, or Adjuvant! Online sites. It will be important to cater this to patients rather than physicians, since physicians will have stronger biases, I think. - This needs to simplified, as even QALYs may be too complex. People might rather see the difference in recurrence risk and % complications, maybe, shown on a time line/graph? This will depict the matter of QALYs perhaps. - Calculations of comorbidity will be important for the model since the results are so dependent on life expectancy, this could be a difficult part of having success with the model. - Need to explain QALYs and utilities to patients, will be challenging. - Suggest plotting life expectancy and risks of side effects. - For genomics test, the field is kind of split regarding whether we should consider these genomics tests to be valid at this point. - Showing the base case is confusing to patients, don’t show the average. - This is reminiscent of Talcott’s work, since disease recurrence prevention has QoL benefits. - QALYs will be a foreign concept to patients, should also show clinical outcomes on graphs. - Simple graphs are best for communication, no need to compare to average. - Think about adding Charlson score to estimating mortality from other causes. - Think about comparing rates of death from prostate cancer to death from other causes.	- I am excited about the Decipher test too but is it ready to be incorporated as a required tool in the model? It has not been proven prospectively to be useful in the adjuvant/salvage decision. - Since it takes a month to get results, it is not practical to incorporate for a first time consult, face to face discussion. - I think it’s too complex to integrate with integrate with EMR at this point. - This could be very useful clinically, with a dual audience (patients and providers). - This should be available direct to patient. - This is definitely something patients would like. There should be a patient web portal, and then the patient can review it with doctors after studying it. It may help to have clinicians present this to patient first.
Biomedical Informatics (*n* = 2)	None.	None.	- The EMR is challenging for collecting and incorporating individual data. - Design: do not need to show comparison of average values to patients, this would be too confusing. - A lot of EMRs are trying to allow add-ons to EPIC. If that is done, this could interact with MyChart. - Self-rated health question could be used to estimate mortality.	- Can pull age and medical comorbidities from EMR. - Need to figure out how report gets entered into EMR. - Genetic counselor could input report into EMR. - The doctor/patient pair in an office is a good way to deliver information. - This needs to be accessible with communication, so need to promote this to patients. - Midlevels (e.g., NPs) in urology could discuss and input data for this.
Genomic Assay Industry Representatives (*n* = 5)		- Using a test ID code could be used to de-identify this. - Would need to ensure PHI secure, may be able to use test codes that don’t link to PHI.	- This is useful for patients to see it plotted graphically. Would be nice to give patients control to play around with inputs and numbers. - Both graphs and numbers are important. - Would want to be able to print both graph and numbers. - Really need to keep individual factors simple, and make sure it’s easy to enter the Decipher score. - For a given test score, this show’s what is risk given variety of treatment scenarios. - A random code can be assigned to a patient’s report to input Decipher score in web portal. - Important that model is accurate; need to continually update inputs based on evidence (like MSKCC nomograms).	- It’s important that this be discussed with physician and patient together. - EMR linkage may help control accuracy. May want to make this accessible both patient-controlled and also in EMR. - Need to create an independent website and include disclaimer that this is for research use only (like nomograms). - Can potentially store information in one place and link to research database. - This might be a commercially viable type of system—selling point for businesses to use this; could do per-click or contract with companies. - Offices who order Decipher send letter to patients. Some people request copies directly to patient. - Web-based approach seems important.

Content is organized according to stakeholder group and thematic category. *ADT* = androgen deprivation therapy; *CD* = compact disk; *EMR* = electronic medical record; *MD* = medical doctor; *MSKCC* = Memorial Sloan Kettering Cancer Center; *NP* = nurse practitioner; *PHI* = personal health information; *QALYs* = quality-adjusted life years; *QoL* = quality of life

Stakeholders were enthusiastic about the potential value of using decision analysis modeling with personalized model inputs for risk of cancer recurrence and other patient-specific factors such as age, other medical problems and lifestyle. Table 2 displays key decision aid elements that were identified based on stakeholder engagement that will be used to design a more effective patient-centered tool.

**Table 2:** Key elements and themes identified during stakeholder engagement meetings that will inform the development of a personalized decision aid program. QALYs = quality-adjusted life years

Personalized decision aid program: key design element from stakeholder engagement meetings	
• Web portal available for patients and providers to access decision aid report at any time or place • Decision aid needs to be accessible on computers and mobile devices and easily printable • Focus on anonymous data structures that are not automatically linked to electronic medical records • Interactive model that allows patients and clinicians to iteratively adjust model assumptions and observe the impact on model reports • Personalized model inputs to include cancer recurrence (based on genomic classifier test), age, other medical problems, and lifestyle • Short tables of bullet points to provide clinical context for the decision aid subject, to alleviate anxiety, to clarify difference between cancer genomics and human genetic code, and to explain how the personalized model provides a patient-centered framework to contemplate genomic testing results • Simple figures and graphs to communicate differences in estimated QALYs as well as risks of treatment-related toxicities, metastasis and death from prostate cancer versus other causes • The length of the primary report will be limited, to minimize information overload, and model design details and average (base-case) model results will be relegated to an Additional file 1 document for those who are interested	

## Discussion

We engaged a total of 22 stakeholders, including patients, physicians, biomedical informatics faculty members, and genomic testing industry representatives, to gain perspectives and insights regarding our stated goal of developing a framework to deliver personalized decision aids to assist in the shared decision making process for patients making ART and/or SRT decisions with individualized GC-based recurrence risk estimates. Stakeholders were enthusiastic about the general concept of personalized decision aids based upon a heuristic model of Markov decision analysis modeling using several individualized model inputs for event probabilities. Stakeholders provided valuable insights regarding optimal presentation of model results for the decision aid, which will directly influence the design of the decision aid. Stakeholder insights, summarized in Tables 1 and 2, provided valuable guidance regarding decision aid content and design as well as delivery format. Based on stakeholder input, our group plans to move forward with development of a personalized decision aid program that aims to support a patient-centered approach to communicating genomic classifier test results and guiding shared decision-making for cancer treatment decisions.

Physicians recognize genomic testing as a valuable tool for clinical care [26, 27], but substantial challenges remain regarding effectively communicating test results to patients and making clinical decisions that represent both genomic information and patient preferences for treatment [5, 27, 28]. Patients value genomic testing results [29] but, understandably, report substantial confusion regarding the value of gene expression profiling for cancer treatment decisions [4], suggesting that decision aids may be of value to better communicate GC results and help inform appropriate decision-making. Our stakeholder engagement study provides valuable insights into how to effectively communicate GC results in a patient-centered approach [7]. Stakeholders provided guidance on important features of a personalized decision aid program, with recommendations to: include a range of individualized inputs in the Markov model, present results in simple figure forms, allow for an interactive model design, keep genomic data anonymous, streamline results as much as possible, and make the decision aid available on mobile devices and in clinical situations. Integration of genomic information into the electronic health record for clinical use raises myriad challenges and opportunities, so stakeholder input is important when planning successful integration with electronic medical records in the clinical setting [30].

Although many of the insights from stakeholders shown in Table 1 may be generalizable to other clinical situations where genomic test results provide personalized estimates of cancer recurrence, the relevance of some stakeholder comments may be limited to proposed decision aid programs that employ an analytic approach based upon decision analysis modeling like the one developed by our group. It is important to note that all members of our patient stakeholder group reported at least a college degree, and most members had a graduate degree, so our study may fail to fully reflect the broader population. In particular, our finding that there were not privacy concerns among our stakeholders likely reflects the participants’ ability to readily distinguish genomic testing of tumor from genetic testing. It is likely that many members of the public would not have such clear understanding of tumor genomic tests versus genetic tests of disease risks, and that privacy concerns and fear of discrimination based on genetic testing likely remain of significant concern for other patients. The current study is also limited by the lack of inclusion of family members or primary care providers as stakeholders, since both groups have been shown to influence the treatment preferences of prostate cancer patients [31]. Furthermore, we did not engage representatives from insurance companies, so our study does not include the perspective of primary payers. Our findings do not address an emerging genomic test that provides direct predictions regarding response to postoperative radiation therapy [32], a development that could provide more clinically applicable information and reduce the degree of need for a personalized decision aid program. The relatively small sample sizes of stakeholders in the current study presents a substantial limitation of the current study, since the selective sample of participants limits the generalizability of our findings to other populations or clinical situations. Furthermore, the decision to interview some stakeholders, such as patients, individually and to interview other stakeholders, such as physicians and industry representatives, in a group setting may introduce problems in the comparability of findings across stakeholder groups. However, intergroup comparisons were not a major focus of the current study.

## Conclusions

In conclusion, our stakeholder engagement process included perspectives from a range of stakeholders and provided valuable information that will inform the design of a personalized decision aid program for genomic-based treatment decisions regarding ART and SRT after prostatectomy. The guidance provided by our stakeholders may be broadly applicable to the communication of genomic test results to patients in a patient-centered fashion that supports effective shared decision-making.

## Additional file

Additional file 1: Stakeholder Engagement Meeting Slide Set. Slides used to facilitate stakeholder discussions, provided before and during stakeholder engagement meetings. (PDF 496 kb)

## Abbreviations

ART: adjuvant radiation therapy
ASTRO: American Society for Radiation Oncology
AUA: American Urological Association
GC: genomic classifier
PSA: prostate specific antigen
QALYs: quality-adjusted life years
RP: radical prostatectomy
RT: radiation therapy
SRT: salvage radiation therapy
