# Supplementary material for: Providing guidance for genomics-based cancer treatment decisions: insights from stakeholder engagement for post-prostatectomy radiation therapy
Source: BMC Med Inform Decis Mak. 2017 Aug 24;17:128. doi: 10.1186/s12911-017-0526-1 (PMC5571582; doi:10.1186/s12911-017-0526-1)
Supplement: Additional file 1: — Stakeholder Engagement Meeting Slide Set. Slides used to facilitate stakeholder discussions, provided before and during stakeholder engagement meetings. (PDF 496 kb) [file 12911_2017_526_MOESM1_ESM.pdf]

<u>Stakeholder engagement</u> plan for development of personalized decision aid program for prostate cancer treatment decisions after radical prostatectomy

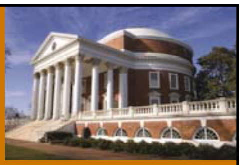

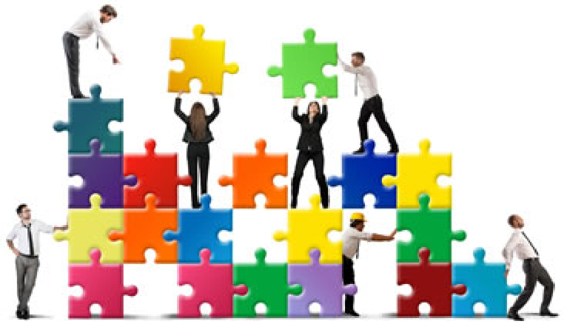

Timothy N. Showalter, MD, MPH

Associate Professor

UNIVERSITY of VIRGINIA HEALTH SYSTEM

Department of Radiation Oncology

What is the goal of this prostate cancer research program?

- Personalized "decision aid" for patients faced with treatment choices
- Clinical perspective for genomic testing that predicts risk of cancer spread

Department of Radiation Oncology

UNIVERSITY of VIRGINIA HEALTH SYSTEM

What is a stakeholder engagement?

- "Meaningful involvement of patients, caregivers, clinicians, and other healthcare stakeholders throughout the research process..."
- "…such engagement can **influence research to be more patient centered**, useful, and trustworthy and ultimately lead to **greater use** and uptake of research results by the patient and broader healthcare community."

\- Patient Centered Outcomes Research Instit

What is the purpose of this meeting today?

- You have been identified as a stakeholder for this topic
- We value your input on the project
- Your input will be recorded in notes
- *Informed consent must be documented*

The Clinical Decision: Immediate vs Delayed Radiation Therapy after Prostatectomy

Post-Operative Radiation Therapy (RT)

- Adjuvant radiation therapy (ART):
  - Negative PSA + concerning pathology findings after surgery
  - ART is directed to the prostate bed to prevent recurrence
- Observation with PSA testing, use delayed salvage RT (SRT) only if/when PSA rises
  - SRT targets the prostate bed to treat PSA-detected microscopic recurrence
  - RT is more effective when PSA is low

Department of Radiation Oncology

University of Virginia Health System

Adjuvant Radiation Therapy

PROS (+)	CONS (-)
High level of evidence (randomized, controlled trials)	Overtreatment. Many men (~40%) would never recur anyways.
Earlier RT may be more effective for some med with highest-risk tumors	Risks of complications applies to all men treated.
Curative. Cure prevents need for hormone therapy and other treatments.	Costs and time. RT is expensive. 7 weeks of daily treatments.

Department *of* Radiation Oncology

University of Virginia Health System

Salvage Radiation Therapy

PROS (+)	CONS (-)
Multiple sources of evidence (not randomized, controlled trials)	Delayed RT may worsen outcomes for men with aggressive tumors.
Fewer patients treated overall.	Risks of complications.
Curative. Cure = no need for hormone therapy and other treatments.	Costs and time. RT is expensive. 7 weeks of daily treatments.

Department *of* Radiation Oncology

University of Virginia Health System

Adjuvant and Salvage Radiotherapy After Prostatectomy: AUA/ASTRO Guideline

Ian M. Thompson,* Richard K. Valicenti,* Peter Albertsen, Brian J. Davis, S. Larry Goldenberg, Carol Hahn, Eric Klein, Jeff Michalski, Mack Roach, Oliver Sartor, J. Stuart Wolf, Jr. and Martha M. Faraday

From the American Urological Association Education and Research, Inc., Linthicum, Maryland, and the American Society for Radiation Oncology, Fairfax, Virginia

"…the patient, his family and the multi-disciplinary treatment team should engage in a **shared decision-making process** in which the patient is advised to consider the possibility of additional treatment (i.e. radiotherapy). Whether ART should be administered is a decision best made by the multidisciplinary treatment team and the patient with consideration of the patient's history, functional status, values, preferences and tolerance for the potential toxicities and QoL effects of radiotherapy"

Department of Radiation Oncology

University of Virginia Health System

Personal Factors to Consider

- Priorities: aggressive treatment vs. quality of life
- Level of concern for side effects
- Opinions of family and friends
- Physician recommendations (and bias)
- Life expectancy and other medical problems
- Genomic tests: new way to estimate individual risk of cancer spread

Department of Radiation Oncology

University of Virginia Health System

Genomic Classifier Test

- Genomic test validated for post-prostatectomy RT decisions
  - Personalized risk of cancer spread
  - A molecular "thumbprint" of the cancer
- This genomic test has been shown to influence physician recommendations

Department of Radiation Oncology

University of Virginia Health System

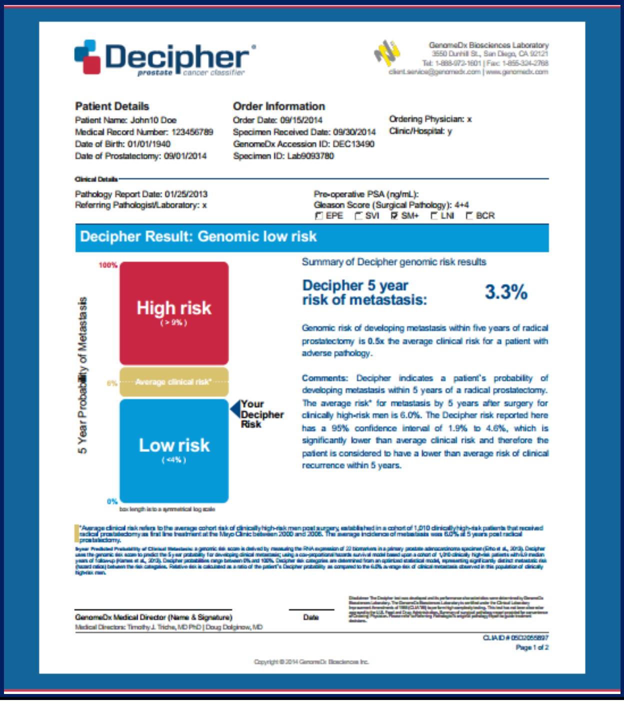

Decipher® prostate cancer classifier

GenomeDx Biosciences Laboratory
3550 Dunhill St., San Diego, CA 92121
Tel: 1-888-972-1601 | Fax: 1-855-324-2768
client.service@genomedx.com | www.genomedx.com

Patient Details
Patient Name: John10 Doe
Medical Record Number: 123456789
Date of Birth: 01/01/1940
Date of Prostatectomy: 09/01/2014

Order Information
Order Date: 09/15/2014
Specimen Received Date: 09/30/2014
GenomeDx Accession ID: DEC13490
Specimen ID: Lab9093780

Ordering Physician: x
Clinic/Hospital: y

Clinical Details
Pathology Report Date: 01/25/2013
Referring Pathologist/Laboratory: x
Pre-operative PSA (ng/mL):
Gleason Score (Surgical Pathology): 4+4
☐ EPE ☐ SVI ☑ SM+ ☐ LNI ☐ BCR

Decipher Result: Genomic low risk

Summary of Decipher genomic risk results

Decipher 5 year risk of metastasis: 3.3%

Genomic risk of developing metastasis within five years of radical prostatectomy is 0.5x the average clinical risk for a patient with adverse pathology.

Comments: Decipher indicates a patient's probability of developing metastasis within 5 years of a radical prostatectomy. The average risk* for metastasis by 5 years after surgery for clinically high-risk men is 6.0%. The Decipher risk reported here has a 95% confidence interval of 1.9% to 4.6%, which is significantly lower than average clinical risk and therefore the patient is considered to have a lower than average risk of clinical recurrence within 5 years.

*Average clinical risk refers to the average cohort risk of clinically high-risk men post surgery, established in a cohort of 1,010 clinically high-risk patients that received radical prostatectomy as first line treatment at the Mayo Clinic between 2000 and 2006. The average incidence of metastasis was 6.0% at 5 years post radical prostatectomy.

[illegible]

GenomeDx Medical Director (Name & Signature) Date
Medical Directors: Timothy J. Triche, MD PhD | Doug Dolginow, MD

[illegible]

CLIA ID # 05D2055897
Page 1 of 2

Copyright © 2014 GenomeDx Biosciences Inc.

Complex Decision for RT following RP

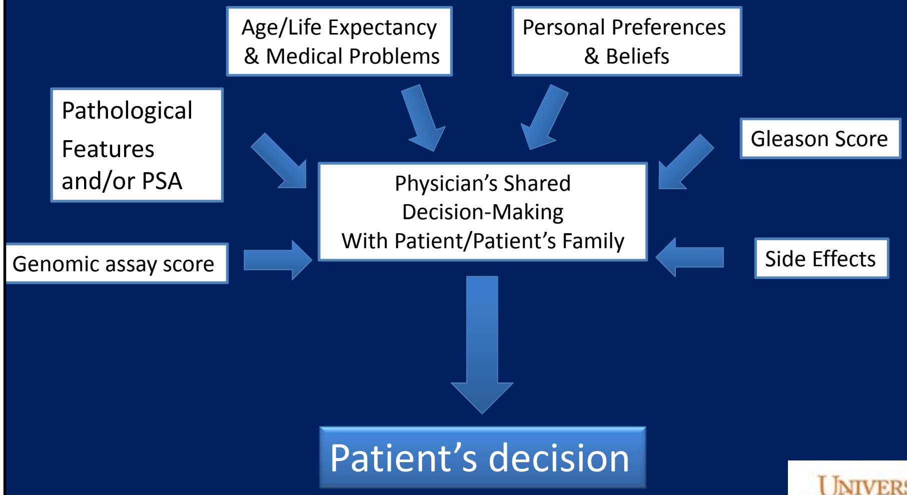

Department of Radiation Oncology

What are the potential opportunities to improve decision making?

- *Our Current Strategy*: Research from grant from American Society for Radiation Oncology (ASTRO)
- Aim 1) Perform decision analysis modeling to evaluate the comparative effectiveness of adjuvant and salvage therapeutic strategies after prostatectomy for locally advanced prostate cancer. Model simulates effects of individual patient's age, preferences, and genomic test results.
- Aim 2) Create a decision support tool to guide personalized, shared decision making regarding adjuvant and salvage therapies after prostatectomy for patients with locally advanced prostate cancer.

Department of Radiation Oncology

Decision Analysis Modeling (Aim 1)

- Markov model of Adjuvant RT vs. Observation/Salvage RT
  - 10-year time horizon.
- Clinical health states, possible complications, and "utilities" in model.
- Utility is a value that represents an individual patient's preference for a particular health state, with potential values ranging from 0 (death) to 1 (perfect health).
- Model results: quality adjusted life-years (QALYs)

Department of Radiation Oncology

University of Virginia Health System

Transition State Diagram

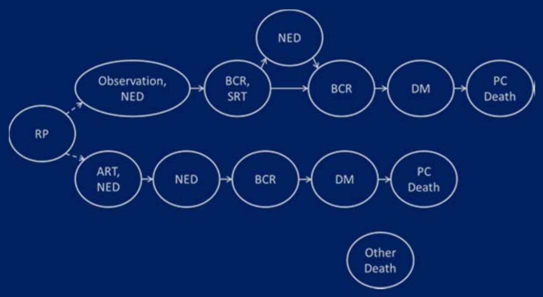

This figure shows the overall structure of the Markov model. After prostatectomy (RP), half of the simulated patients are assigned to Observation and half to adjuvant radiation therapy (ART). In monthly cycles over a 10-year time horizon, transitions occur from left to right at a specified probability based on risk of prostate cancer recurrence. In the model, each transition has associated impact on treatments received, side effects of treatment, and changes in utility values. The model also includes a continuous age-based risk of death from other causes (Other Death).

Department of Radiation Oncology

University of Virginia Health System

Model Inputs

- Published literature for average values
- Individualized-range inputs designed to simulate wide differences among patients:
  - Age-adjusted mortality risk: based on life tables maintained by US governmental agency
  - Genomic classifier score
  - Utilities: full reported range
  - Complication risks over wide range

Department of Radiation Oncology

University of Virginia Health System

Overall Findings

- On average, Observation/SRT (8.1 QALYs) preferred over ART (6.73 QALYs).
- Observation/SRT favored even when model inputs varied dramatically (including utilities).
- However, genomic-based risk changes results:

HIGH RISK Genomic Score

- ART strongly preferred (~80% of paired sample runs).

LOW RISK Genomic Score

- Observation/SRT strongly preferred (98% of paired sample runs).

Factors that Alter Optimal Treatment

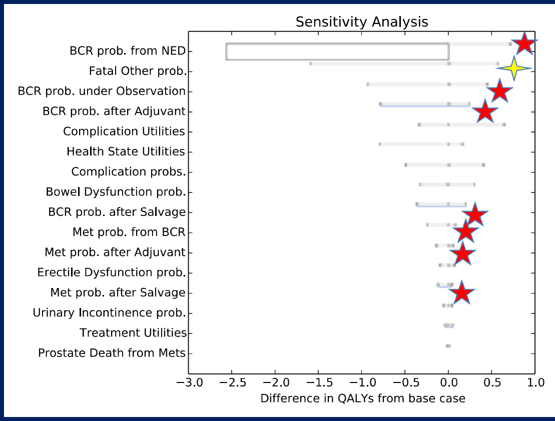

- ★ = from genomic test score
- ✦ = based on patient age

*All other factors were based upon the best average estimates published in literature, with sensitivity analyses based on broadest range:

- Utilities were lowest and higher reported values
- Complication probabilities from ½ to 2x average

Department of Radiation Oncology

UNIVERSITY of VIRGINIA HEALTH SYSTEM

Our Modeling Conclusions

- Genomic test results most influential in model
- Age-based risk of death is also important
  - Opportunity to refine/adjust methods
- Across broad range of values, utility inputs (patient preferences for outcomes) had relatively small effect on outcomes
- Genomics-based recurrence inputs creates new opportunity for this approach to be used to provide personalized reports

Department of Radiation Oncology

UNIVERSITY of VIRGINIA HEALTH SYSTEM

Leveraging Model Results for Personalized Decision Aid

- We plan to develop a decision aid program that uses Markov modeling to provide personalized guidance for adjuvant vs observation decisions based on individualized data for:
  - genomic test result
  - Patient age +/- additional comorbidities
- A simplified pictorial approach will be used for reporting

Department of Radiation Oncology

University of Virginia Health System

Hypothetical Decision Aid Format

Personalized Input 1

Patient Age: __ years old

Personalized Input 2

Genomic Test Result: __% risk at 5 years

Personalized Input 3

Major Medical Problems: [listed from chart]

__% risk of mortality

Sample Report (HIGH Genomic Score)

Patients with a similar clinical presentation as you have had **significantly more** quality adjusted life years (QALYs) with adjuvant therapy than observation

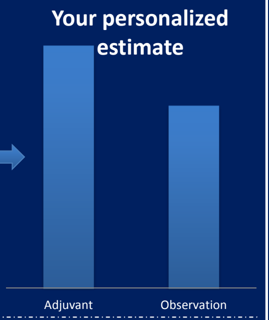

Additional text and graphs to describe risk and projected outcomes based on personalized Markov model results

Department of Radiation Oncology

University of Virginia Health System

Feedback

- Is this a useful addition to genomics info?
- How should information be presented?
  - Numbers vs. general themes
  - Graphs/pictures vs. numerical format
- Where should this be available?
  - Only in MD office?
  - Direct to patient beforehand?

Department of Radiation Oncology

University of Virginia Health System

What are potential barriers?

- Who would patients want to discuss this with (MD or nurse)?
- How can we make this be understandable?
- Would web-based portal be accessible?
- Is more information needed to provide a helpful guide?

Department of Radiation Oncology

University of Virginia Health System
